# First-in-human phase I clinical trial of a recombinant vesicular stomatitis virus (rVSV)-based preventive HIV-1 vaccine

**DOI:** 10.1186/1742-4690-9-S2-P134

**Published:** 2012-09-13

**Authors:** JD Fuchs, I Frank, N Kochar, M Elizaga, M Allen, D Carter, N Frahm, SA Kalams, M Mulligan, R Sheets, M Pensiero, D Clarke, J Eldridge

**Affiliations:** 1 San Francisco Dept. of Public Health, San Francisco, CA, USA; 2 University of Pennsylvania, USA; 3 Fred Hutchinson Cancer Center, USA; 4 Division of AIDS, NIH, USA; 5 Vanderbilt University, USA; 6 Emory University, USA; 7 Profectus Biosciences, USA

## Background

Replicating viral vectors are promising HIV vaccine candidates that may enhance immunogenicity through prolonged antigen expression. VSV is the first replicating viral vector after vaccinia to be tested clinically as an HIV vaccine; here we present preliminary safety and immunogenicity data from a phase 1a trial.

## Methods

HVTN 090 enrolled sixty healthy, HIV-1-uninfected adults in a randomized, double-blinded, placebo-controlled dose escalation study. Groups of 12 participants received rVSV Indiana HIV Gag vaccine at 5 dose levels (4.6 x 10^3^ to 3.4 x 10^7^ PFU) (N=10/group) or placebo (N=2/group), delivered intramuscularly at 0 and 2 months. Reactogenicity over 7 days, adverse events (AEs), and viral cultures from whole blood, urine, saliva and swabs of oral lesions were collected. HIV-1-specific CD4+ and CD8+ T-cell responses to Gag peptides were measured 1 and 2 weeks post-boost by intracellular cytokine staining.

## Results

The study is ongoing and data are blinded. The median age was 24; 47% were female and 37% were non-white. Local and systemic reactogenicity was self-limited, mild to moderate in intensity and increased with dose, with headache reported most commonly (52%). At the highest dose, 92% reported systemic symptoms, including flu-like syndrome (41%), fever (41%), and moderate chills (33%). Lymphadenopathy, decreased neutrophil count, oral ulceration, and presyncope were each seen in > 1 participant. No severe reactogenicity, encephalitis, or product-related SAEs were reported, and all VSV cultures were negative at all doses tested. Low frequency HIV-specific CD4+ (9%) and CD8+ (3%) T-cell responses were detected post-boost at the first 3 dose levels.

## Conclusion

Immunization with an attenuated, replicating rVSV Indiana HIV-1 vaccine has an acceptable reactogencity and safety profile to date. Preliminary data reveal few T-cell responses at lower doses. Immunogenicity of the vaccine at the highest doses and in heterologous prime-boost regimens will guide future vector development.

